# Stopping runaway seizures with a chill pill

**DOI:** 10.7554/eLife.102055

**Published:** 2024-09-05

**Authors:** Gordon F Buchanan

**Affiliations:** 1 University of Iowa, Carver College of Medicine Iowa City United States

**Keywords:** epilepsy, seizures, status epilepticus, hypothermia, neurotensin, Mouse

## Abstract

The neuropeptide neurotensin can reduce status epilepticus and its associated consequences through induction of therapeutic hypothermia when bound to a molecule that can penetrate the blood-brain barrier.

**Related research article** Ferhat L, Soussi R, Masse M, Kyriatzis G, Girard SD, Gassiot F, Gaudin N, Laurencin M, Bernard A, Bôle A, Ferraci G, Smirnova M, Roman F, Dive V, Cisternino S, Temsamani J, David M, Lécorché P, Jacquot G, Khrestchatisky M. 2024. A peptide-neurotensin conjugate that crosses the blood-brain barrier induces pharmacological hypothermia associated with anticonvulsant, neuroprotective and anti-inflammatory properties following status epilepticus in mice. *eLife* **13**:RP100527. doi: 10.7554/eLife.100527.

The brain consists of electrically excitable cells called neurons, which work in concert to regulate brain function. Sometimes, these cells become aberrantly connected and may activate together as a small network, leading to abnormal bursts of electrical activity known as seizures. Seizures disrupt the flow of messages between neurons and can lead to involuntary changes in behavior, sensation, and body movement or function.

Recurrent seizures are the hallmark of disease in patients with epilepsy (Langbein et al., 2024). Seizures that last a long time, also known as status epilepticus, can be life-threatening and require immediate treatment. Unfortunately, status epilepticus is often very difficult to control, leading to a high rate of morbidity and mortality (Kämppi et al., 2024).

Efforts to develop better therapies for status epilepticus are ongoing (O’Kula and Hill, 2024; Kishihara et al., 2024). One way to manage these kinds of seizures involves inducing hypothermia in the body and the brain to reduce cellular excitability (Kirmani et al., 2021). However, lowering body temperature using physical means, such as cooling blankets or infusions of cooled saline, can negatively affect the body. A more targeted way would be through medication that could pharmacologically alter body temperature.

While drugs targeting the biochemical pathways that regulate temperature homeostasis can lower body temperature, they often fail to reach the brain due to the blood-brain barrier, a protective semipermeable membrane between the blood and the brain. Thus, many drugs that lower body temperature do not markedly reduce brain temperature and have little to no effect on status epilepticus. Now, in eLife, Lotfi Ferhat, Michel Khrestchatisky and colleagues report a new approach to specifically reduce brain temperature and stop status epilepticus (Ferhat et al., 2024).

The researchers, who are based at various research institutes in France, targeted the biochemical pathway involving the signaling molecule neurotensin, which is known to regulate body temperature by lowering the set point on the thermostat that governs body temperature. As neurotensin cannot cross the blood-brain barrier, Ferhat et al. combined neurotensin with a molecule known to penetrate the brain and tested its effectiveness in mice. Specifically, these ‘conjugated’ versions were able to bind the low-density lipoprotein receptor (LDLR), which then transported the molecule across the blood-brain barrier intothe brain.

The researchers developed several conjugate proteins, and through a series of careful testing, identified one to employ throughout the study. The conjugate formed by this protein and neurotensin was successful in reaching the brain, inducing hypothermia, and reducing the seizures and cognitive and inflammatory changes associated with status epilepticus. However, it still needs to be confirmed that neurotensin-induced hypothermia alone is responsible for alleviating the effects associated with status epilepticus.

Regardless, the possibility of pharmacologically induced hypothermia for managing status epilepticus is exciting. Refractory status epilepticus (which continues despite treatment) is a dreadful condition with extremely high rates of mortality, despite the barrage of currently available therapies. Improved treatment modalities are sorely needed. While the findings of Ferhat et al. are promising, more needs to be learned about the safety of these treatments for the rest of the body. Such studies could be first trialled in animals but would later need to be replicated in humans. Moreover, while it is assumed that pharmacological means to induce hypothermia would have fewer negative consequences than physical means, this needs to be empirically evaluated. Hypothermia itself can have profound negative consequences, so the degree of hypothermia would need to be tightly monitored (Lu et al., 2024).

The extent of neuroprotection afforded by pharmacologically induced hypothermia could likely extend to other forms of neural injury, including stroke, post-anoxic brain injury or traumatic brain injury (Lin et al., 2024). Thus, implications for this promising work are potentially extremely far-reaching.
